# Dung beetles response to livestock management in three different regional contexts

**DOI:** 10.1038/s41598-020-60575-5

**Published:** 2020-02-28

**Authors:** Celeste Beatriz Guerra Alonso, Gustavo Andrés Zurita, M. Isabel Bellocq

**Affiliations:** 1 Instituto de Biología Subtropical, Universidad Nacional de Misiones-CONICET Puerto Iguazú, Misiones, Argentina; 2 Facultad de Ciencias Forestales, Universidad Nacional de Misiones-CONICET, Eldorado, Misiones, Argentina; 3 Departamento de Ecología, Genética y Evolución, Facultad de Ciencias Exactas y Naturales, Universidad de Buenos Aires, Buenos Aires, Argentina

**Keywords:** Biodiversity, Community ecology, Forest ecology, Conservation biology

## Abstract

The response of biological communities to human disturbances depends on factors acting at local and regional scale and on the interaction between them. We compared the response of native forest dung beetle communities to cattle grazing under regional contexts differing on precipitation patterns (Atlantic forest and humid and dry Chaco). Through multivariate and GLMM analyses we contrasted richness and composition across regions and land uses and explored the role of local and regional variables accounting for those changes. We captured a total of 44101 individuals of 109 species. The interaction between local and regional variables influenced the response to livestock management. In the two wet regions (humid Chaco and Atlantic forest) diversity was similar in the native forest regardless of cattle presence but differs strongly in open pastures. In contrast, in the dry Chaco, differences between native forest and land use were not evident. Vegetation structure was a major determinant of species richness, whereas regional climate determined differences in species composition. We concluded that the response of dung beetles to livestock management cannot be generalized for all biomes. In dry ecosystems, dung beetles are probably pre-adapted to environmental conditions imposed by cattle ranching whereas in wet ecosystems the impact of cattle ranching is more significant.

## Introduction

Biological diversity can be described at local and regional scale^1,2^. At a local scale, abiotic factors act as environmental filters preventing or allowing the establishment and/or persistence of species^3^. Hierarchically structured ecological filters select a sub-sample of species from the regional pool^4–7^. This filtering process is usually not random; in local communities, species sharing functional traits are grouped under certain environmental conditions^4^. At larger scales, latitudinal, longitudinal or altitudinal gradients of temperature and/or precipitation also act as ecological filters influencing regional patterns of diversity^8,9^. The relationship between large-scale climate and diversity has been described for a number of taxa, including terrestrial plants^10^, vertebrates^11^ and insects^12^. Previous studies in tropical and subtropical areas have shown that precipitation (particularly seasonal precipitation) is usually the strongest predictor of diversity regional patterns (e.g.^9,13–15^).

The replacement of native forests by grazing areas is one of the main causes of the global biodiversity crisis^16^. This process reduces the number of species in biological communities and tends to homogenize the composition of species throughout different regions and environments (also called homogenization process)^17^. Consequently, from an ecological perspective, the extirpation of species as a result of human disturbances can be considered a novel ecological filter^18,19^; in addition, human disturbance can influence the condition of existing ecological filters (such as resources availability). Previous studies have shown that the response of populations and communities to human disturbance partially depends on the similarity between the native and the disturbed habitat^20,21^. Land uses maintaining key components from the native habitat, such as specific resources or abiotic conditions, usually preserve the conditions of ecological filters and the diversity of species, whereas land uses that strongly change the filter conditions are used mainly by extra-regional or invasive species with different ecological requirements^20,22–24^. While different land uses within a region can be viewed as a gradient of suitability for native species^25^, the intensity of these changes can be strongly influenced by the regional context or biome as the species’ range of tolerance to environmental conditions, which defines the ecological niche, at this scale depends on evolutionary processes^24^. In general, the regional context or biome sets the species’ range of tolerance to environmental conditions (ecological niche width)^26,27^.

*Scarabaeinae* dung beetles are widely used as focal taxa in ecological studies because of their high diversity, wide distributional ranges, ecological role and sensitivity to human disturbances^28,29^. Previous studies have shown a reduction in both the taxonomic and functional diversity of dung beetles associated to the replacement of native forests by open pastures for cattle grazing^30–32^. In contrast, a series of recent studies showed that cattle areas preserving the forest canopy (particularly of native trees) totally or partially preserve the native diversity of dung beetles in forest ecosystems^33–37^. Livestock systems preserving the canopy also maintain microclimatic conditions and part of the native forest vegetation structure^36,37^. Canopy cover has an indirect influence on dung beetles through the maintenance of soil and understory microclimatic conditions (temperature and humidity)^38^. Considering that forest dung beetles are characterized by a low tolerance to extreme microclimatic conditions^39–42^, disturbances altering microclimatic factors directly affect forest species^39–41,43^.

Although the response of local patterns of dung beetles communities to forest replacement by cattle areas has been described and conservation recommendations at this scale are clear, studies focusing at regional scale are scarce^30,34^. Biodiversity conservation at a large scale requires studies that focus on regional patterns and the potential mechanisms influencing large patterns of diversity^44–46^. Moreover, the two scales interact to determine the response of communities to human disturbances^23,24^. Our objective is to compare the response of dung beetle assemblages to similar land uses (cattle grazing in open pastures and native forests) under dissimilar regional contexts differing mainly on precipitation patterns (both seasonality and total amount). Under the hypothesis that both local and regional factors influence the diversity of dung beetle communities to cattle grazing, we expect a stronger response in land uses and regions exhibiting a higher contrast between grazing areas and the native forest.

## Methods

### Experimental design

#### Study area

**Figure 1:**
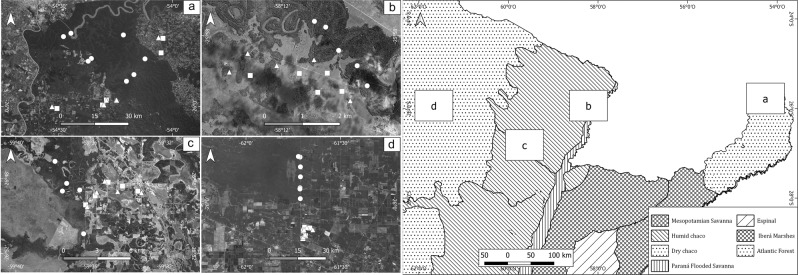
Sampling areas in three subtropical forests of North Argentina (right): (**a**) Atlantic forest; (**b**) and (**c**) humid Chaco; (**d**) dry Chaco. In the detailed figure (left), native forest (circles), silvopastoral systems (triangles) and open pastures (squares). Maps were created using ArcGIS software v10.7 by Esri. ArcGIS and ArcMap are the intellectual property of Esri and are used herein under license. Copyright © Esri.

This study was performed in three seasonal Neotropical dry forest domains (SNDFD) of South America^47^ (Fig. 1): the southern Atlantic forest, the humid Chaco and the dry Chaco. Sampling areas on each region were located at a similar latitude (between 25 °58′S − 26 °48′S), with similar patterns of temperature, mainly differing in total amount and seasonal precipitation patterns (Table 1)^48–51^. In order to increase the temporal representation, each region was sampled in two consecutive years (2015–2017) during the spring (October-December), the season with the highest dung beetle activity in tropical and subtropical regions^52^. For a detailed description of sampling sites see Supplementary material Table S1.

**Table 1:** Environmental description of the three sampled regions in the subtropics of Argentina.

	Atlantic Forest	Humid Chaco	Dry Chaco
Climate	Warm and humid	Temperate humid	Warm
Temperature °C (annual average)	20 °C	22 °C	23 °C
Precipitation (mm)	1600–2000	750–1300	500–700
Precipitation Seasonality	Low	Concentrated in spring-summer (October to April)	Concentrated in spring-summer (October to April)
Vegetation	Homogenous, dense canopy cover	Highly heterogeneous, forming a complex mosaic of forest, grassland and wetland	Mosaic of xeric forest and grassland
Altitude (m)	270	75	158

In each region and year, we established five replicates of three environments: (1) native forests without cattle (native forest: NF), (2) native forests with cattle (silvopastoral system: SS), and (3) open grasslands with cattle (open pastures: OP). Replicates within each region were separated by at least 1000 m to ensure they would not affect on each other.

#### Dung beetle collection

To capture dung beetles, we installed 10 pitfall traps separated 50 m from each other in each replicate (three regions x two years x three environments x five replicates x 10 traps = 450 traps/year). Traps consisted of a plastic container (12 cm in diameter and depth) filled with 200 ml of water, neutral detergent and salt to avoid the decomposition of individuals without interfering with attraction^41^. We baited traps with approximately 20 g of human faeces and rotten meat (five traps with each bait) to attract both coprophagous and necrophagous dung beetles^53^. We collected the beetles and renewed the bait in three consecutive periods (8 sampling days) every 48 hours. Traps within each replicate were considered (10) as sub-samples and added for data analysis^54^. We identified the species through consultation with specialists, taxonomic keys^55^, and comparison with a reference collection of the study area (IBSI Sca). Collected individuals were deposited in the *Scarabaeidae* Collection of the Instituto de Biología Subtropical - Iguazú (IBSI Sca), Misiones, Argentina.

#### Environmental description at local and regional scales

To describe vegetation structure at the local scale, we established three 5 ×15 m sub-plots on each replicate of each environment and region (three plots x three regions x three environments x five replicates = 135 plots). We averaged sub-plots within each replicate to obtain a single value. In each sub-plot, we estimated four variables based on a scale of abundance-coverage (0–100%): (1) bare soil; (2) herbaceous vegetation; (3) shrub vegetation; and (4) canopy cover. Additionally, to determine litter cover, we collected three samples of litter from a 50 × 50 cm quadrant within the plot, they were dried in a stove at 70 °C for 72 hours and then weighed. Automatic temperature and humidity sensors (HOBO U23002) were installed in all sites to record temperature and humidity at the ground level, every five minutes throughout the sampling period. Then, we averaged temperature and humidity to obtain a single value per site. We calculated thermal amplitude by subtracting the minimum from the maximum daily temperature. Finally, we calculated the average daily maximum temperature.

At a regional scale, for each replicate of each environment and region we selected three bioclimatic variables from the WorldClim dataset^56^ widely used on regional studies (e.g.^57^): (1) BIO1 = Annual Mean Temperature, (2) BIO2 = Mean Diurnal Range (Mean of monthly (max temp - min temp)) and (3) BIO15 = Precipitation Seasonality (Coefficient of Variation). Variables represent an average for the period 1970–2000 with a spatial resolution of 30 seconds (~1 km^2^).

### Data analysis

To explore the completeness of the sampling effort on each region and environment we first calculated the SC estimator using the iNEXT SC^58^.

### Patterns of richness: local vs. regional factors

To compare richness at both scales (and the interaction between scales), we performed a mixed generalized linear model using region and environment as fixed factors and the interaction between both factors. We grouped sampling year and area into a single factor and included it in the model as a random factor. We assumed a Poisson distribution of errors (discrete variable) and we related richness to the set of predictive variables (environment and region) through a logarithmic link function using the glmer function in R (nlme4 package)^59^. Finally, we compared the model with the null model to determine the significance of individual factors. We evaluated normality and homoscedasticity through residuals vs. predicted plots and qqnorm; in addition, we evaluated overdispersion.

To evaluate the assumption of no spatial autocorrelation on GLMM analysis of richness, we calculated the Moran’s I index^60^ as a global measure of spatial autocorrelation for the residuals of our regression model using SAM v4.0^61^. In this analysis we used eleven distance classes, with a size equal number of pairs, which maximized the similarity in the number of observations among classes. Then, we evaluated the statistical significance of a deviation from 0 (no spatial pattern). Since the residuals of the regression model for richness were not significantly autocorrelated (see Supplementary Fig. S1), the geographical coordinates of sampling sites were not included in GLMM analysis.

To explore the role of local and regional environmental variables explaining patterns of species richness, we first performed three independent PCA to reduce the number of explanatory variables: (1) one with local vegetation structure (cover of canopy, litter, tree, shrubs, herbs and bare soil); (2) one with local microclimatic conditions (thermal amplitude, average daily temperature and humidity, and average daily maximum temperature); and (3) one with regional climate (annual mean temperature, mean diurnal range, precipitation seasonality). Then, we used the first axis of each PCA in a GLMM model to explain differences in dung beetle richness among environments and regions. We evaluated normality and homoscedasticity through residuals vs. predicted and qqnorm plots; we also evaluated overdispersion. Finally, we compared the model with the null model to determine the significance of individual factors. We evaluated collinearity among the first axis of each PCA (predictor variables of the model) through vif function of car package^62^.

### Patterns of beta diversity: local vs. regional factors

To explore changes in species composition among environments and regions, and the relation with local and regional environmental variables, we first evaluated the spatial structure of dung beetle assemblage composition (C) through the partition of the variation. In this analysis, we used the Bray-Curtis index of dissimilarity calculated with the varpart function in the vegan package^63^. The partition of the variation discriminates the percentage of influence of environmental variables (E) from the spatial structure (S) (and the combined influence of E|S)^64^. This method estimates and tests the percentage of variation (*r*^2^ adj) attributed to each unique set of explanatory variables. Finally, we estimated the significance of each component through permutation tests (N = 9999)^65^.

**Figure 2:**
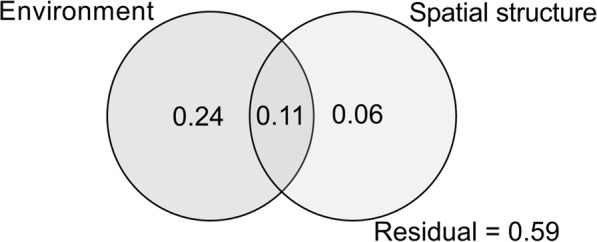
Influence (proportion of the explained variance) of environmental variables and spatial structure explaining patterns of dung beetle assemblage composition among native forests, silvopastoral systems and open pastures in three subtropical forest of Argentina (Atlantic forest and humid and dry Chaco). The residual represents the proportion of the total variation that was not explained by these variables.

To determine the effect of environmental variables on dung beetles’ species composition we performed a db-RDA (distance-based redundancy analysis)^66^. Since in the previous analysis (partition of the variation) the spatial data structure had only a small influence on species composition (6%) (see Fig. 2; Table 2), we excluded spatial structure from this analysis. We performed this analysis through the permutation-based ANOVA (with 9999 permutations) in the vegan package^63^. Through a stepwise procedure, the db-RDA analysis determines the influence of individual environmental variables. We then selected the most parsimonious model based on the Akaike information criterion (AIC) and tested it on a 9999 permutation analysis. Before the analysis, we standardized environmental variables and root square transformed to reduce the impact of extreme outliers^67^. Environmental variables exhibiting multicollinearity (>0.6) were excluded from the analysis. Finally, we tested the explanatory power of the region, the environment, and also the interaction between these two factors in the groups formed by db-RDA; to do this, we performed a permutational multivariate analysis of variance (PERMANOVA), using the adonis function of the vegan package^63^. We performed all statistical analyses in R^68^.

**Table 2:** Influence of environmental variables and spatial structure in a partition of variation analysis on spatial patterns of dung beetle composition in three subtropical forests of Argentina (Atlantic forest, humid and dry Chaco).

	d.f.	Adj. *R*^2^	F	*P*-value
Environment	6	0.35	8.93	0.001
Spatial	2	0.17	10.07	0.001
All	8	0.41		
Environment|Spatial	6	0.24	6.86	0.001
Spatial|Environment	2	0.06	5.21	0.001
Residuals		0.59		

To explore the influence of human land use on dung beetles species composition, we calculated 1-the quantitate Jaccard index of similarity (beta diversity, dissimilarity) between native undisturbed dung beetles communities in forest without cattle and those on cattle areas in each region (SS and PA) using the BAT package in R^69^. Finally, as in previous analysis, we compared the dissimilarity among land uses and regions using a GLMM analysis with sampling year and area as random factors, assuming a normal distribution.

## Results

The total number of individuals collected in all environments (Native forest-NF, Silvopastoral system-SS, Open pastures-OP) and regions (Atlantic forest, humid and dry Chaco) was 44101, belonging to 109 species (Supplementary Table S2). A total of 50 species were collected in the Atlantic forest, 55 in the humid Chaco and 46 in the dry Chaco. Sampling coverage was above 0.98 in all cases, showing that the sampling effort was enough to capture most of the species (Supplementary Table S2). In the native forest and the silvopastoral systems of the Atlantic forest and the humid Chaco, the most captured species was *Canthon quinquemaculatus*. In the dry Chaco most of the collected individuals in the native forest were *Deltochilum variolosum* and in the silvopastoral system, *Onthophagus* aff. *hircus*. In open pastures, *Eutrichilum hirsutum* and *Dichotomius nisus* were the most numerous species captured in the Atlantic forest, *Deltochilum elongatum* in the humid Chaco and *Malagoniella puncticollis* in the dry Chaco (Supplementary Table S2).

### Patterns of richness: local vs. regional factors

**Figure 3:**
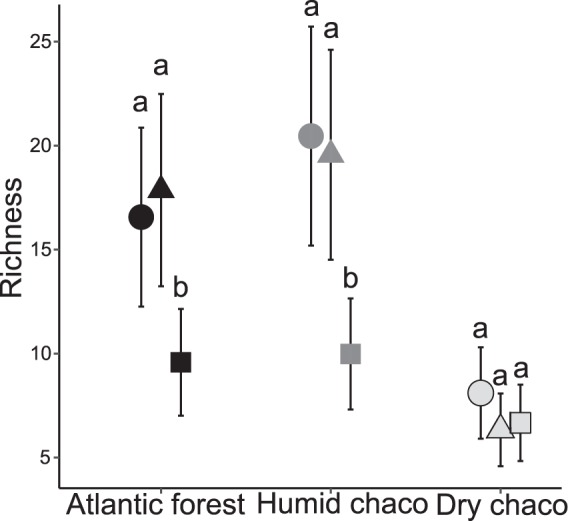
Richness of dung beetle assemblages in native forest and livestock systems in subtropical forests of Argentina. Circles: Native forest; triangles: Silvopastoral systems; squares: Open Pastures. Different letters indicate significant differences (P < 0.05).

The GLMM analysis (r^2^ = 0.691, Chisq = 80.78, P < 0.0001) revealed that dung beetles richness differed from regions (Chisq = 6.27, P = 0.043), environments (Chisq = 52.46, P = <0.0001) and the interaction between them (Chisq = 15.95, P = 0.003) (Supplementary Results S1 online). In the Atlantic forest and the humid Chaco, richness was similar in NF and SS, and lower in OP. In the dry Chaco, richness was similar in the three environments (Fig. 3).

The first axis of the three PCA performed to reduce the number of explanatory variables with (1) local vegetation structure, (2) local microclimatic conditions and (3) regional climate, explained more than 50% of the variation in all cases (Supplementary Results S2 online). The GLMM analysis with richness using the first axis of those PCA explained 35.6% of the variation in the number of species and showed that dung beetle richness was mainly explained by local vegetation structure (Chisq = 19.6, P < 0.001), whereas local microclimatic conditions and regional climate had no influence on it (Chisq = 0.76 and Chisq = 0.004, respectively, P > 0.1 in both cases).

### Patterns of beta diversity: local vs. regional factors

As showed in Fig. 2 and Table 2 (partition of the variation analysis), environmental variables explained 35% (24% after controlling by spatial structure) of the observed variation in dung beetle assemblage composition among environments and regions, whereas spatial structure explained 17% (6% after controlling by environmental variables). The combined influence of environmental variables and spatial structure explained 11% of the variation, whereas 59% of the variation was not explained by the model (residuals).

**Figure 4:**
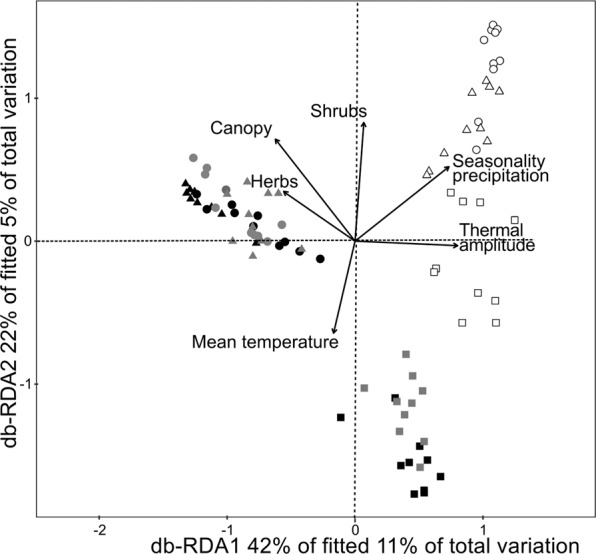
Distance-based redundancy analysis (db-RDA) based on the composition of dung beetle assemblages in the native forest and two livestock systems in subtropical forests of Argentina. Black symbols: Atlantic forest; gray symbols: humid Chaco; white symbols: dry Chaco. Circles: native forest; triangles: silvopastoral system; squares: open pastures.

Figure 4 shows the results from the db-RDA analysis (C~E); the first axis explained 42% of changes in dung beetle composition among regions and environments. On this axis, NF and SS from the Atlantic forest and the humid Chaco formed a single group and separated from the dry Chaco. The second axis explained 22% of the variation and separated the OP of the Atlantic forest and the humid Chaco from the other sites (Fig. 4). The PERMANOVA analysis validated these groups (F model = 9.99, r^2^ = 0.56, d.f. = 10, P = 0.001); sampling sites separated according to the region (F = 19.62, r^2^ = 0.23, d.f. = 2, P = 0.001), the environment (PERMANOVA, F = 11.37, r^2^ = 0.13, d.f. = 2, P = 0.001) and the interaction between region and environment (PERMANOVA, F = 6.12, r^2^ = 0.15, d.f. = 4, P = 0.001).

On the db-RDA analysis (Table 3 and Fig. 4), environmental variables explained 26.7% of dung beetle composition changes among environments and regions (d.f. = 6, F = 6.09, P = 0.001). The first axis was negatively associated to canopy and herbaceous cover and positively associated to precipitation seasonality and thermal amplitude, whereas the second axis was positively correlated with shrub cover and negatively correlated with mean temperature. The NF and SS in the Atlantic forest and the humid Chaco were mainly associated to higher canopy and herbaceous cover, and showed a higher abundance of *Canthon quinquemaculatus* (r = −0.64), *Coprophanaeus cyanescens* (r = −0.55) and *Deltochilum* aff. *komareki* (r = −0.53). The OP located in the Atlantic forest and the humid Chaco were mainly associated to a higher local average temperature and a higher abundance of *Deltochilum elongatum* (r = −0.26) and *Dichotomius nisus* (r = −0.61). Finally, sites in the dry Chaco were mainly associated to a higher seasonality of precipitation and to thermal amplitude, and a higher abundance of *Deltochilum variolosum* (r = 0.52).

**Table 3:** Role of local (L) and regional (R) environmental variables in a db-RDA explaining patterns of dung beetle assemblage composition among the native forest and two livestock systems (silvopastoral and open pastures) in subtropical forests of Argentina (Atlantic forest, humid and dry Chaco). r^2^adj: adjusted coefficient of determination, d.f.: degrees of freedom: 1, Corr.: Correlation.

	Axis 1 Corr.	Axis 2 Corr.	*r*^*2*^ adj	F	*P*-value
Precipitation seasonality (R)	0.682	0.494	0.073	8.00	0.002
Canopy cover (%) (L)	−0.579	0.676	0.133	7.10	0.002
Shrub cover (%) (L)	0.064	0.787	0.162	4.	0.002
Average temperature (°C) (L)	−0.156	−0.611	0.180	2.80	0.002
Thermal amplitude (°C) (L)	0.745	−0.029	0.200	3.13	0.002
Herbaceous cover (%) (L)	−0.511	0.323	0.214	2.53	0.002

**Figure 5:**
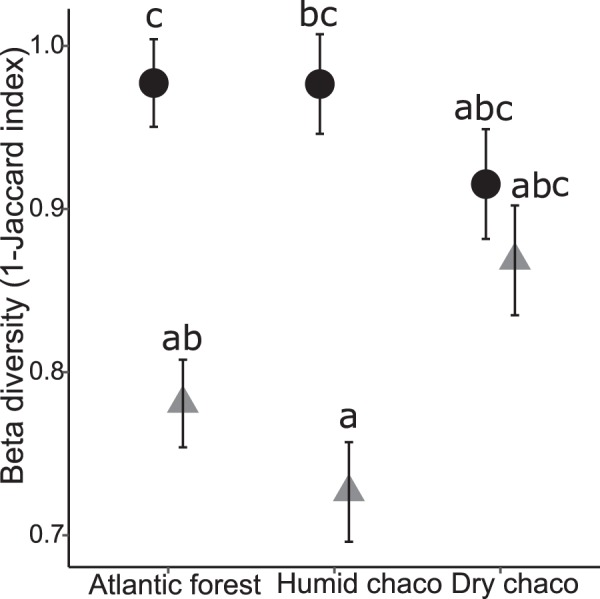
Dissimilarity (1-Jaccard index) on species composition between the native undisturbed forest and cattle areas in subtropical forests of Argentina. White symbols: native forest vs. open pasture, gray symbols: native forest vs. silvopastoral system, bars: standard error. Different letters indicate significant differences (P < 0.05).

Finally, Fig. 5 shows the results of the dissimilarity analysis (β diversity: 1-Jaccard index) between the native dung beetles community of each region and that of cattle areas (SS and PA). Similar to previous results, the GLMM analysis showed that, in both the humid Chaco and the Atlantic forest the dissimilarity with the native forest community was greater in OP compared to SS whereas in the dry Chaco both OP and SS showed similar values in relation to the native forest (Region: F = 0.53, P = 0.631; Environment: F = 204.47, P < 0.001; Region*Environment: F = 12.16, P < 0.001).

## Discussion

Our hypothesis was that the response of dung beetle assemblages to livestock management depends not only on factors acting at local and regional scales but also on the interaction between both scales. Our results support the hypothesis: the interaction between the region and the environment influenced both the richness and the composition of dung beetle assemblages. In the two wet regions with a low-intermediate precipitation seasonality (the humid Chaco and the Atlantic forest), dung beetle diversity in open livestock areas strongly differed from the native forest and the silvopastoral system; in contrast, in the region showing the strongest seasonality of precipitation (dry Chaco) differences on dung beetle diversity between livestock systems and native forests were not evident. Most previous studies have focused on the local consequences of cattle grazing^31–33,35,41,70,71^ or on the importance of regional and local factors determining patterns of dung beetle diversity^30,72–74^. However, this is one of the first field studies that explicitly considers the interaction of both scales through the comparison of the response of dung beetles to cattle raising among different regions.

As shown in previous studies, replacement of the native forest by open pastures strongly reduced dung beetle richness and modified species composition in the Atlantic forest^31,35,75^ and the humid Chaco^37,76^; in addition, in both regions silvopastoral systems preserved dung beetle diversity. In contrast, dung beetle assemblages in the dry Chaco showed a completely different response: diversity (both richness and composition) was similar in open pastures, native forests and silvopastoral systems. While this is the first study conducted with dung beetles in this dry ecosystem the differential response probably reflects the environmental similarity between native forest and grazing areas in the dry Chaco. Environmental dissimilarity among native environments and land uses has been already identified as one of the main predictors of changes on biological communities at both local and regional scales^23,24^. In particular, previous studies with dung beetles have shown that land uses preserving microclimatic conditions at the ground level, understory vegetation and soil structure maintain a greater number of native species than those that drastically change them^34,35,37,41,71^. In both the humid Chaco and the Atlantic forest, open pastures contrast greatly with the native forest in vegetation structure and microclimatic conditions, whereas in the dry Chaco environmental conditions between the native environment and open cattle pastures were more similar.

Although this is the first study conducted in the dry Chaco, the response of forest dung beetle assemblages to cattle grazing was similar to that in other xeric forests in Mexico^30,77–79^ and Brazil^80^. In these xeric forests, the diversity of dung beetles in grazing areas was even higher than in the native vegetation. These dry forests, which are similar to those of the dry Chaco, exhibit a heterogeneous and complex landscape structure, with areas of shrubs, pastures and forests. Milchunas *et al*.^81^, proposed a model predicting that in semi-arid ecosystems with a long history of herbivore grazing, an increase in grazing pressure results in low (or null) diversity loses compared to more humid ecosystems. Evolutionary physiological adaptations (particularly water stress tolerance) in semi-arid environments may facilitate the use of open pastures in grazing areas by native species^82^. Larsen^83^ proposed that species in dry ecosystems are more tolerant to land use changes due to a broader range of physiological tolerance which evolved as a response to extreme daily fluctuations in temperature. Also, species in xeric ecosystems show a lower respiratory water loss rate, which allows them to tolerate extreme conditions^39,42^. In addition, the reduced competition in arid environments (as a consequence of communities with fewer species) may allow species to evolve wider ecological niches. Finally, and similar to our results in the dry Chaco, disturbed and undisturbed habitats in dry ecosystems show, in general, similar microclimatic conditions for dung beetles^83^.

Previous studies showed that changes on environmental conditions (as a consequence of human disturbances) are highly dependent on the regional context^20,84^. Regional climatic conditions determine the distributional range of dung beetles, both in their native range and in recently introduced areas^85^. Davis *et al*.^86^ performed a multiscale analysis and concluded that, at a regional scale, diversity of dung beetles increases with average annual temperature and precipitation, and decreases with soil stoniness. On the other hand, aridity (like in the dry Chaco) was associated to less diverse assemblages composed mainly by species active primarily under cold and humid conditions after rainfall events. Also, in a recent study, Liu *et al*.^87^ determine that abundance and composition of several functional groups of beetles (herbivores, predators and decomposers) respond mainly to regional factors, despite land use intensity and landscape context. Additionally, Jacobs *et al*.^88^ showed that in South African mosaics of grassland and forest (with a climate similar to that of the dry Chaco), vegetation ecotypes were the major determinant of species composition, independently of current land uses. Despite the few studies dealing with the joint effect of climatic factors at regional and local scales, evidence from this and previous studies strongly suggests that, in dry environments, the response of assemblages to land use is better explained by climatic conditions.

We showed that the response of dung beetle assemblages to livestock management cannot be generalized for all biomes: the evolutionary history of assemblages strongly influences their response to human land uses. Factors acting at local and regional scales interact to produce different spatial patterns of assemblages response to human land uses. From a conservation and economic perspective and considering the central role dung beetles play on the organic matter cycle in cattle areas, the regional context should be considered to evaluate the impact of human land uses on biodiversity and ecosystem functioning. Moreover, our results show that in dry ecosystems dung beetles are probably pre-adapted to environmental conditions imposed by cattle ranching, whereas in wet ecosystems cattle ranching has a strong impact on dung beetles.

## Supplementary information

Supplementary Information.

## Data Availability

Richness and abundance data per region and environment are available in Table S2 (Supplementary Information). Climatic data were downloaded http://worldclim.org/version2. Replicates location of the environments within each region, are available in Table S1 (Supplementary Information).
