# Supplementary material for: Dung beetles response to livestock management in three different regional contexts
Source: Sci Rep. 2020 Feb 28;10:3702. doi: 10.1038/s41598-020-60575-5 (PMC7048846; doi:10.1038/s41598-020-60575-5)
Supplement: Supplementary file 1 — Supplementary Information. [file 41598_2020_60575_MOESM1_ESM.pdf]

Supplementary Information

Dung beetles response to livestock management in three different regional contexts

Celeste Beatriz Guerra Alonso1*, Gustavo Andrés Zurita 1,2, M. Isabel Bellocq 3†

(1) Instituto de Biología Subtropical, Universidad Nacional de Misiones-CONICET Puerto Iguazú, Misiones, Argentina

(2) Facultad de Ciencias Forestales, Universidad Nacional de Misiones-CONICET, Eldorado, Misiones, Argentina

(3) Departamento de Ecología, Genética y Evolución, Facultad de Ciencias Exactas y Naturales, Universidad de Buenos Aires, Buenos Aires, Argentina

* Corresponding author

E-mail: clstguerra@gmail.com

Table S1: Description of sampling sites in the Atlantic forest, Humid Chaco and Dry Chaco of Argentina.

Region	Geographical location	Sampling time	Forest native	Silvopastoral system	Open pasture
Atlantic forest	between 25°58'2″ S, 54°17′22″W and 26°36′32″S, 54°41′43″W	13-20 October 2015	1) 25° 40' 38"S, 54° 26' 46"W	26° 00' 04"S, 54° 31' 48"W	26° 00' 32"S, 54° 30' 28"W
			2) 25° 41' 32"S, 54° 28' 45"W	25° 58' 21"S, 54° 14' 40"W	25° 58' 17"S, 54° 17' 24"W
			3) 25° 47' 19"S, 54° 21' 23"W	25° 58' 22"S, 54° 17' 26"W	25° 59' 37"S, 54° 18' 24"W
			4) 25° 48' 02"S, 54° 22' 12"W	25° 59' 36"S, 54° 18' 02"W	25° 45' 50"S, 54° 03' 00"W
			5) 25° 51' 34"S, 54° 10' 07"W	25° 41' 46"S, 54° 03' 38"W	25° 42' 00"S, 54° 02' 29"W
		12-19 December 2016	1) 25° 53' 19"S, 54° 12' 06"W	25° 41' 53"S, 54° 03' 35"W	25° 42' 02"S, 54° 02' 25"W
			2) 25° 47' 25"S, 54° 07' 12"W	25° 58' 17"S, 54° 14' 47"W	25° 45' 52"S, 54° 03' 00"W
			3) 25° 41' 06"S, 54° 12' 54"W	25° 58' 20"S, 54° 17' 23"W	25° 58' 19"S, 54° 17' 23"W
			4) 25° 47' 19"S, 54° 21' 19"W	25° 59' 35"S, 54° 18' 15"W	25° 59' 39"S, 54° 18' 18"W
			5) 25° 48' 02"S, 54° 22' 12"W	26° 00' 06"S, 54° 31' 41"W	26° 00' 31"S, 54° 30' 25"W

Humid Chaco	North samplings (between 25º 59' 24.2″S, 58º10'48″ W and 25°57'33.6″S, 58°11'27.1″W)	29 October to 6 November 2015	1) 25° 57' 45"S, 58° 11' 27"W<br>2) 25° 58' 47"S, 58° 10' 37'W'<br>3) 25° 58' 13"S, 58° 11' 04"W<br>4) 25° 58' 28"S, 58° 10' 46"W<br>5) 25° 57' 51"S, 58° 11' 16"W	25° 58' 28'S', 58° 12' 03"W<br>25° 58' 16"S, 58° 12' 30'W'<br>58' 34"S, 58° 12' 48"W<br>25° 58' 22"S, 58° 11' 27"W<br>25° 59' 01"S, 58° 10' 53"W	25° 58' 35"S, 58° 11' 41"W<br>25° 58' 37"S, 58° 12' 28"W<br>25° 58' 53"S, 58° 11' 24"W<br>25° 58' 35"S, 58° 11' 15"W<br>25° 58' 52"S, 58° 11' 01"W
	South samplings (between 26°46'52.7″S, 59º37'2.8″ W and 26°50'6.6″S, 59°36'17.6″W)	10-18 November 2016	1) 26° 48' 36"S, 59° 36' 36"W<br>2) 26° 48' 15"S, 59° 37' 18"W<br>3) 26° 47' 04'S, 59° 37' 55"W<br>4) 26° 48' 40"S, 59° 37' 31"W<br>5) 26° 50' 57"S, 59° 36' 23"W	26° 48' 44"S, 59° 35' 41"W<br>26° 48' 08"S, 59° 35' 15"W<br>26° 48' 42"S, 59° 34' 33"W<br>26° 48' 45"S, 59° 33' 17"W<br>26° 49' 30"S, 59° 36' 11"W	26° 48' 12"S, 59° 35' 04"W<br>26° 48' 24"S, 59° 34' 18"W<br>26° 48' 40"S, 59° 33' 28"W<br>26° 49' 24"S, 59° 36' 16"W<br>26° 48' 56"S 59° 35' 46"W

Dry Chaco	between 25°44'52.8″S, 61°43'33.6″W and 26°11'11.4″S, 61°42'19.6″W	31 October to 7 November 2016	1) 25° 45' 07"S, 61° 42' 52"W<br>2) 25° 48' 14"S, 61° 42' 43"W<br>3) 25° 51' 24"S, 61° 42' 43"W<br>4) 25° 55' 16"S, 61° 42' 53"W<br>5) 25° 58' 21"S, 61° 42' 46"W	26° 09' 44"S, 61° 41' 09"W<br>26° 08' 13"S, 61° 41' 35"W<br>26° 07' 33"S, 61° 40' 39"W<br>26° 08' 13"S, 61° 38' 47"W<br>26° 10' 30"S, 61° 41' 53"W	26° 10' 06"S, 61° 41' 08"W<br>26° 08' 36"S, 61° 41' 50"W<br>26° 07' 28"S, 61° 41' 26"W<br>26° 07' 45"S, 61° 39' 47"W<br>26° 11' 02"S, 61° 41' 58"W
		9-17 October 2017	1) 25° 45' 07"S, 61° 42' 50"W<br>2) 25° 44' 56"S, 61° 43' 23"W<br>3) 25° 54' 54"S, 61° 42' 50"W<br>4) 25° 55' 16"S, 61° 42' 53"W<br>5) 25° 58' 21"S, 61° 42' 46"W	26° 09' 44"S, 61° 41' 11"W<br>26° 08' 13"S, 61° 41' 35"W<br>26° 07' 33"S, 61° 40' 39"W<br>26° 08' 13"S, 61° 38' 47"W<br>26° 10' 30"S, 61° 41' 51"W	26° 10' 09"S, 61° 41' 07"W<br>26° 08' 31"S, 61° 41' 53"W<br>26° 07' 23"S, 61° 41' 28"W<br>26° 07' 48"S, 61° 39' 50"W<br>26° 11' 02"S, 61° 41' 56"W

Autocorrelogram

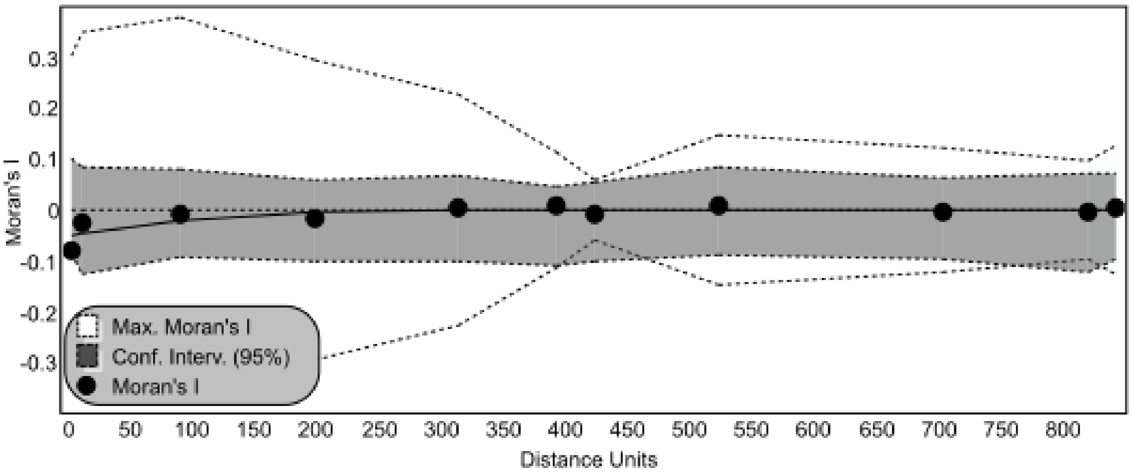

Fig. S1: Autocorrelogram showing the Moran's index of spatial autocorrelation for the richness values of model residuals. Significance was tested using 9,999 permutations. Black circles indicate non-significant correlations.

Table S2: Number of individuals captured and richness of dung beetles in the native forest and two livestock systems in subtropical forests of Argentina. N: total number of collected individuals; S. obs: number of species; SC: sample coverage estimator; NF: native forest; SS: silvopastoral system; OP: open pasture.

	Atlantic forest						Humid chaco						Dry chaco					
	**2015**			**2016**			**2015**			**2016**			**2016**			**2017**		
**Species**	**NF**	**SS**	**OP**	**NF**	**SS**	**OP**	**NF**	**SS**	**OP**	**NF**	**SS**	**OP**	**NF**	**SS**	**OP**	**NF**	**SS**	**OP**
Anisocanthon sp.	0	0	0	0	0	0	110	34	0	93	55	0	0	0	0	0	0	0
Ateuchus robustus	0	0	0	0	0	0	1	3	0	0	0	0	0	0	0	0	0	0
Ateuchus sp.	0	0	0	0	2	0	0	0	0	0	0	0	0	1	0	0	0	0
Atheucus ovalis	0	0	0	0	0	0	0	0	0	0	0	0	4	0	0	0	0	0
Bolbites onitoides	0	0	0	0	0	0	0	0	4	0	0	38	0	0	1	0	0	0
Canthidium aff. trinodosum	0	3	0	0	0	0	0	0	0	0	0	0	0	0	0	0	0	0
Canthidium bituberculatum	0	2	1	0	3	4	0	0	0	0	0	0	0	0	0	0	0	0

Canthidium boquermani	0	0	0	0	0	0	0	0	0	0	0	0	0	1	2	0	0	0
Canthidium breve	0	0	0	0	0	0	0	0	0	0	1	0	155	29	13	3	1	0
Canthidium cavifrons	0	1	0	0	3	0	0	0	0	0	0	0	0	0	0	0	0	0
Canthidium cuprinum	0	0	0	0	0	0	1241	134	0	15	0	0	0	0	0	0	0	0
Canthidium dispar	3	1	0	3	0	0	0	0	0	0	0	0	0	0	0	0	0	0
Canthidium hyla	1	3	4	1	0	1	0	0	0	0	0	0	0	0	0	0	0	0
Canthidium lucidum	0	0	0	0	1	0	0	0	0	0	0	0	0	0	0	0	0	0
Canthidium nobile	0	0	0	0	4	0	0	0	0	0	0	0	0	0	0	0	0	0

Canthidium prasinum	0	0	0	0	0	0	0	0	0	0	0	0	0	0	11	0	0	0
Canthidium sp. 1	0	0	0	0	0	0	0	2	0	0	0	0	0	0	0	0	0	0
Canthidium sp. 2	0	0	0	0	0	0	0	4	0	0	2	0	13	0	0	1	3	0
Canthidium sp. 2	34	8	0	25	12	0	539	32	1	143	184	0	1	0	0	0	0	0
Canthon aff. chalybaeus	0	0	0	0	0	0	0	0	0	30	49	0	0	0	0	0	0	0
Canthon aff. lituratus	0	0	0	0	0	0	0	0	15	0	0	0	0	0	0	0	0	0
Canthon aff. mutabilis	0	0	158	0	0	0	0	0	8	0	0	0	0	0	0	0	0	0
Canthon aff. muticus	0	0	0	0	0	0	0	0	0	0	7	0	0	0	0	0	0	0
Canthon aff.	0	0	0	0	0	0	0	0	0	0	0	0	1	1	0	0	0	0

piluliformis																		
Canthon aff. podagricus	0	0	0	0	0	2	0	0	0	0	0	0	0	0	0	0	0	0
Canthon bipuntatus	0	0	0	0	0	0	0	0	0	0	0	0	58	2	3	19	0	1
Canthon bispinus	0	0	0	0	0	0	0	24	18	0	0	383	0	0	0	0	0	0
Canthon chiriguano	0	1	0	0	2	0	4	0	0	7	0	0	0	0	0	0	0	0
Canthon conformis	7	111	9	1	473	15	19	1	2	156	17	0	0	0	0	0	0	0
Canthon curvodilatatus	0	0	10	0	0	0	1	136	0	0	1	22	19	5	9	0	0	0
Canthon daguerrei	0	0	0	0	0	0	0	35	1	0	0	156	0	0	0	0	0	0

Canthon denticulatus	0	0	0	0	0	0	1	0	0	0	0	1	0	0	0	0	0	0
Canthon deplanatus	0	0	0	0	0	0	0	0	0	0	0	0	0	0	2	0	0	0
Canthon histrio	2	142	0	1	422	2	2490	213	0	31	0	0	0	0	0	0	0	0
Canthon lituratus	0	0	0	0	0	0	0	0	0	0	0	0	0	0	4	0	0	0
Canthon maldonadoi	0	0	0	0	0	0	1	0	2	37	3	0	0	8	1	1	0	0
Canthon ornatus	0	0	0	0	0	0	0	0	6	0	0	0	0	0	0	0	0	0
Canthon podagricus	0	0	0	0	0	0	0	0	98	0	0	0	0	0	0	0	0	0
Canthon pseudoforcipatus	0	0	0	0	0	0	1	0	0	0	0	0	2	0	0	0	0	0
Canthon	1517	1254	6	279	2248	53	4399	1656	0	1212	702	76	0	8	0	0	1	0

quinquemaculatus																		
Canthon smaragdulus	32	0	0	0	0	0	18	0	0	0	0	0	0	0	0	0	0	0
Canthon sp. 1	0	0	0	0	0	0	0	2	0	0	0	1	0	0	0	0	0	0
Canthon sp. 2	0	0	0	0	0	0	0	0	0	0	0	0	18	8	8	0	0	0
Canthon sp. 3	0	0	0	0	0	0	0	0	0	0	0	0	0	1	0	0	0	0
Canthon sp. 4	0	0	0	0	0	0	0	0	10	0	0	0	0	0	0	0	0	0
Canthon sp. 5	0	0	0	0	0	0	0	0	0	0	0	0	2	4	7	0	0	0
Canthon sp. 6	0	0	0	0	0	0	0	0	0	0	0	0	43	31	2	0	0	0
Canthon sp. 7	0	0	0	0	0	0	0	0	2	3	35	0	23	50	21	0	2	0
Canthon unicolor	0	0	0	0	0	0	92	1321	10	170	480	0	96	57	3	0	0	0
Chalcocopris hesperus	3	0	0	24	0	0	0	0	0	0	0	0	0	0	0	0	0	0

Coprophanaeus bonariensis	0	0	0	0	0	0	0	0	0	0	0	0	4	0	2	0	2	0
Coprophanaeus cyanescens	24	42	9	117	112	8	25	3	25	176	156	1	0	0	0	0	0	0
Coprophanaeus milon	0	0	0	0	0	0	3	1	1	0	0	0	0	0	1	0	0	0
Coprophanaeus saphirinus	190	92	0	69	50	2	0	0	0	0	0	0	0	0	0	0	0	0
Deltochilum aff. komareki	170	630	1	102	630	19	54	0	0	73	17	0	0	0	0	0	0	0
Deltochilum brasiliense	3	2	0	20	0	0	0	0	0	0	0	0	0	0	0	0	0	0
Deltochilum elongatum	0	0	0	0	0	0	15	518	126	158	193	1174	0	0	0	0	0	0
Deltochilum	2	6	0	25	7	0	0	0	0	0	0	0	0	0	0	0	0	0

furcatum																		
Deltochilum icaroides	1	6	0	0	1	0	0	0	0	0	0	0	0	0	0	0	0	0
Deltochilum morbillosum	19	0	0	4	0	0	0	0	0	0	0	0	0	0	0	0	0	0
Deltochilum pseudoicarus	0	0	0	0	0	0	0	0	0	1	1	0	2	0	0	0	0	0
Deltochilum sculpturatum	1	0	0	0	0	0	0	0	0	0	0	0	0	0	0	0	0	0
Deltochilum valgum	0	0	0	0	0	0	0	0	0	1	0	0	0	0	0	0	0	0
Deltochilum variolosum	0	0	0	0	0	0	3	16	0	0	0	33	322	103	41	577	64	39
Dichotomius aff. bicuspis	0	0	0	0	1	0	0	0	0	0	0	0	0	0	0	0	0	0

Dichotomius bitiensis	0	0	0	0	0	0	0	0	0	0	0	0	13	0	0	0	0	0
Dichotomius carbonarius	3	9	7	15	114	3	2808	767	2	59	266	3	5	2	1	0	0	0
Dichotomius depressicollis	16	5	0	4	3	0	0	0	0	0	0	0	0	0	0	0	0	0
Dichotomius fornicatus	0	0	0	0	0	0	0	0	0	0	0	0	37	29	54	0	0	0
Dichotomius micans	0	0	0	0	0	0	0	0	0	0	0	0	2	7	5	0	0	0
Dichotomius mormon	1	1	0	17	30	0	0	0	0	0	0	0	0	0	0	0	0	0
Dichotomius nisus	0	2	148	0	0	133	0	0	111	0	8	0	1	1	48	1	0	59
Dichotomius	0	0	0	0	0	0	0	0	0	0	1	0	0	0	0	0	0	0

nobilis																		
Dichotomius sericeus	50	112	5	250	484	0	0	0	0	107	3	0	0	0	0	0	0	0
Digitonthophagus gazella	0	0	0	0	0	0	0	0	3	0	0	1	0	0	3	0	0	0
Eurysternus aeneus	0	0	1	0	3	0	44	5	1	199	64	0	0	0	0	0	0	0
Eurysternus caribaeus	59	35	0	363	151	0	362	40	0	35	8	0	0	0	0	0	0	0
Eurysternus howdeni	0	0	0	1	0	0	0	0	0	0	0	0	0	0	0	0	0	0
Eurysternus paralellus	332	140	1	132	296	2	0	0	0	0	0	0	0	0	0	0	0	0
Eutrichillum hirsutum	14	22	236	1	46	66	0	0	0	0	0	0	0	0	0	0	0	0

Gromphas inermis	0	0	0	0	0	0	0	0	52	0	0	66	0	0	0	0	0	0
Malagoniella punctatostriatus	0	0	0	0	0	0	1	0	0	3	19	1	1	0	0	0	0	0
Malagoniella puncticollis	0	0	0	0	0	0	0	0	0	0	0	0	156	0	132	2	0	10
Martinezidium galileoae	0	0	0	0	0	0	0	0	0	0	0	0	3	0	0	0	0	0
Nunoidium argentinum	0	0	0	0	0	0	0	0	0	0	0	0	0	0	1	0	0	0
Ontherus apendiculatus	0	0	0	0	0	0	0	0	0	0	0	0	0	1	1	0	0	0
Ontherus erosioides	0	0	0	0	1	0	0	0	0	0	0	0	0	0	0	0	0	0
Ontherus sulcator	2	8	27	0	12	41	633	210	16	11	44	8	1	4	5	1	8	0

Onthophagus aff. buculus	4	2	8	0	2	7	0	0	0	0	0	0	0	0	0	0	0	0
Onthophagus aff. hircus	0	0	0	0	0	0	124	126	0	32	10	0	2	14	2	30	215	1
Onthophagus buculus	0	0	4	0	0	0	0	0	0	0	0	0	0	0	0	0	0	0
Onthophagus catherinensis	116	1	0	55	3	0	0	0	0	0	0	0	0	0	0	0	0	0
Onthophagus sp.	0	0	0	0	0	0	0	0	0	0	0	0	0	0	0	1	0	0
Onthophagus sp. 1	0	0	0	0	0	0	1526	163	0	59	116	0	0	0	0	0	0	0
Onthophagus tristis	2	2	1	20	2	0	55	2	0	0	3	0	0	0	0	0	0	0
Phanaeus splendidulus	3	0	0	2	0	0	0	0	0	0	0	0	0	0	0	0	0	0

Pseudocanthon xanthurus	0	0	0	0	0	0	3	194	0	1	92	0	0	0	0	0	0	0
Scybalocanthon nicriceps	1	0	0	8	0	0	0	0	0	0	0	0	0	0	0	0	0	0
Sulcophanaeus menelas	0	0	0	0	0	0	0	0	0	0	0	0	0	0	1	0	0	0
Trichillidium quadridens	0	0	0	0	0	0	11	5	0	3	187	0	0	6	0	0	0	0
Trichillum epipleuralis	0	0	0	0	1	0	0	0	0	0	0	0	0	0	0	0	0	0
Trichillum hesper	0	0	10	0	0	0	0	0	0	0	0	0	0	0	0	0	0	0
Trichillum externepunctatum	0	0	5	0	0	21	0	0	9	0	1	0	6	9	26	0	0	0
Trichillum sp.	0	0	0	0	0	0	0	0	0	0	0	0	6	0	0	0	0	0

Trichillum sp. 1	0	0	0	0	0	0	0	0	0	0	1	0	0	0	0	0	0	0
Uroxys dilaticollis	11	4	0	1	0	0	0	0	0	0	0	0	0	0	0	0	0	0
Uroxys sp.	1	1	0	2	1	0	0	16	0	0	0	0	2	0	0	0	0	0
Uroxys sp. 1	0	0	0	0	0	0	0	18	0	0	0	0	0	0	0	0	0	0
Vulcanocanthon sp.	0	0	0	0	0	0	0	0	0	0	0	0	1	6	5	0	0	0
**n**	2624	2648	379	1542	5120	651	2815	2726	523	14584	5681	1964	999	388	415	636	296	110
**S.obs**	31	30	16	27	31	20	26	31	23	29	29	15	30	25	30	10	8	5
**SC**	0.9977	0.9977	0.9974	0.9961	0.9988	0.9923	0.9989	0.9978	0.9924	0.9996	0.9996	0.9975	0.994	0.9846	0.9832	0.9921	0.9933	0.9818

Results S1: Mixed generalized linear model selected by hypothesis testing richness

Richness: ~region*environment+ (1|provano),

control=glmerControl(optimizer="bobyqa"), data=Data, family=poisson)

Generalized linear mixed model fit by maximum likelihood (Laplace

Approximation) [glmerMod]

Family: poisson (log)

Formula: Q0 ~ region * environment + (1 | provano)

Control: glmerControl(optimizer = "bobyqa")

AIC	BIC	logLik	deviance	df.resid
491.7	516.7	-235.9	471.7	80

Scaled residuals:

Min	1Q	Median	3Q	Max
-1.8216	-0.6853	-0.1231	0.5812	2.1728

Random effects:

Groups Name Variance Std.Dev.

provano (Intercept) 0.1229 0.3506

Number of obs: 90, groups: provano, 6

Fixed effects:

	Estimate	Std. Error	z value	P value
(Intercept)	2.8072	0.2597	10.808	2.00E-16
regionchaco humedo	0.2112	0.3658	0.578	0.5635
regionchaco seco	-0.7149	0.3755	-1.904	0.0569
ambientepotrero	-0.5476	0.1281	-4.276	1.91E-05
ambientesilvopastoril	0.0754	0.1076	0.7	0.4836
regionchaco humedo:ambientepotrero	-0.1702	0.1768	-0.963	0.3357
regionchaco seco:ambientepotrero	0.3527	0.1985	1.777	0.0756
regionchaco humedo:ambientesilvopastoril	-0.1203	0.1468	-0.819	0.4125
regionchaco seco:ambientesilvopastoril	-0.3223	0.1878	-1.716	0.0861

Correlation of Fixed Effects:

	(Intr)	rchh	rchs	habp	habs	rchh:habp	rchs:habp	rchh:habs
reginchchmd	-0.71							
regionchcsc	-0.692	0.491						
ambientptrr	-0.181	0.128	0.125					
ambntslvpst	-0.215	0.153	0.149	0.436				
rgnchchmd:mbntp	0.131	-0.168	-0.091	-0.724	-0.316			
rgnchcsc:mbntp	0.117	-0.083	-0.22	-0.645	-0.281	0.467		
rgnchchmd:mbnts	0.158	-0.203	-0.109	-0.32	-0.733	0.419	0.206	
rgnchcsc:mbnts	0.123	-0.088	-0.233	-0.25	-0.573	0.181	0.44	0.42

Results S2: Analysis of main components of climate, vegetation and microclimatic conditions.

Eigenvalues, and their contribution to the correlations

Importance of components:

Climate

	PC1	PC2	PC3
Eigenvalue	2.408	0.5472	0.04482
Proportion Explained	0.8027	0.1824	0.01494
Cumulative Proportion	0.8027	0.9851	1

Microclimatic conditions

	PC1	PC2	PC3	PC4
Eigenvalue	2.6736	1.1402	0.14886	0.037273
Proportion Explained	0.6684	0.2851	0.03721	0.009318
Cumulative Proportion	0.6684	0.9535	0.99068	1

Vegetation

	PC1	PC2	PC3	PC4	PC5	PC6
Eigenvalue	3.52	1.1348	0.7126	0.33132	0.24235	0.058905
Proportion Explained	0.5867	0.1891	0.1188	0.05522	0.04039	0.009817
Cumulative Proportion	0.5867	0.7758	0.8946	0.94979	0.99018	1
